# *N*′-(3-Bromo-5-chloro-2-hydroxy­benzyl­idene)-2-chloro­benzohydrazide methanol solvate

**DOI:** 10.1107/S1600536809009647

**Published:** 2009-03-19

**Authors:** Qianfeng Weng, Cunjie Zou

**Affiliations:** a College of Chemistry and Chemical Engineering, Liaoning Normal University, Dalian 116029, People’s Republic of China

## Abstract

In the title compound, C_14_H_9_BrCl_2_N_2_O_2_·CH_4_O, the dihedral angle between the two benzene rings is 49.2 (2)° and an intra­molecular O—H⋯N hydrogen bond occurs. In the crystal struture, mol­ecules are linked by O—H⋯O and N—H⋯O hydrogen bonds.

## Related literature

For related structures, see: Fun *et al.* (2008 ▶); Ali *et al.* (2007 ▶); Zhi & Yang (2007 ▶).
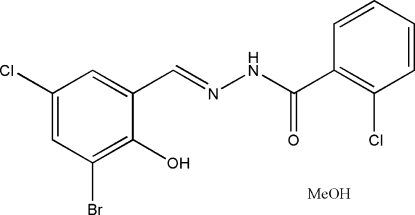


## Experimental

### 

#### Crystal data

C_14_H_9_BrCl_2_N_2_O_2_·CH_4_O
*M* *_r_* = 420.08
Monoclinic, 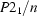

*a* = 11.221 (4) Å
*b* = 9.642 (3) Å
*c* = 15.908 (5) Å
β = 97.537 (5)°
*V* = 1706.3 (10) Å^3^
*Z* = 4
Mo *K*α radiation
μ = 2.74 mm^−1^
*T* = 298 K
0.17 × 0.15 × 0.12 mm

#### Data collection

Bruker SMART 1000 CCD diffractometer
Absorption correction: multi-scan (*SADABS*; Bruker, 2001 ▶) *T*_min_ = 0.653, *T*_max_ = 0.735
9257 measured reflections
3666 independent reflections
2345 reflections with *I* > 2σ(*I*)
*R*_int_ = 0.035

#### Refinement

*R*[*F* ^2^ > 2σ(*F* ^2^)] = 0.039
*wR*(*F* ^2^) = 0.103
*S* = 1.02
3666 reflections
214 parameters
1 restraint
H atoms treated by a mixture of independent and constrained refinement
Δρ_max_ = 0.49 e Å^−3^
Δρ_min_ = −0.46 e Å^−3^

### 

Data collection: *SMART* (Bruker, 2007 ▶); cell refinement: *SAINT* (Bruker, 2007 ▶); data reduction: *SAINT*; program(s) used to solve structure: *SHELXTL* (Sheldrick, 2008 ▶); program(s) used to refine structure: *SHELXTL*; molecular graphics: *SHELXTL*; software used to prepare material for publication: *SHELXTL*.

## Supplementary Material

Crystal structure: contains datablocks global, I. DOI: 10.1107/S1600536809009647/hb2928sup1.cif

Structure factors: contains datablocks I. DOI: 10.1107/S1600536809009647/hb2928Isup2.hkl

Additional supplementary materials:  crystallographic information; 3D view; checkCIF report

## Figures and Tables

**Table 1:** Hydrogen-bond geometry (Å, °)

*D*—H⋯*A*	*D*—H	H⋯*A*	*D*⋯*A*	*D*—H⋯*A*
O1—H1⋯N1	0.82	1.86	2.585 (3)	146
O3—H3⋯O2	0.82	2.04	2.727 (3)	141
N2—H2⋯O3^i^	0.91 (3)	1.93 (3)	2.830 (4)	176 (4)

Symmetry code: (i) 
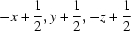
.

**Fig. 1.**
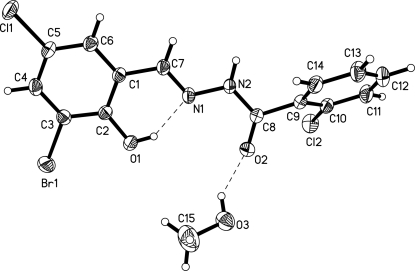
The molecular structure of (I), showing 30% probability displacement ellipsoids for the non-hydrogen atoms. Hydrogen bonds are indicated by dashed lines.

## supplementary crystallographic information

### Comment

Recently, the crystal structures of hydrazone compounds have been widely studied (Fun *et al.*, 2008; Ali *et al.*, 2007; Zhi & Yang, 2007). In this paper, the structure of the title compound, (I), is described.

The title compound consists of a hydrazone molecule and a methanol molecule (Fig. 1). The dihedral angle between the two benzene rings is 49.2 (2)°. The methanol molecule is linked to the hydrazone molecule through an intramolecular O–H···O hydrogen bond (Table 1).

### Experimental

The compound was prepared by the reaction of equimolar quantities (1.0 mmol each) of 3-bromo-5-chloro-2-hydroxybenzaldehyde and 2-chlorobenzohydrazide in methanol (100 ml) for 2 h at room temperature. The solution was kept in air for a week, forming yellow blocks of (I).

### Refinement

The N-bound H atom was located in a difference Fourier map and was refined with an N–H distance restraint of 0.90 (1) Å. Other H atoms were placed in calculated positions (C–H = 0.93–0.96 Å, O–H = 0.82 Å) and refined using a riding model with *U*_iso_(H) = 1.2*U*_eq_(C) and 1.5*U*_eq_(O and C15).

### Crystal data

**Table:**

C_14_H_9_BrCl_2_N_2_O_2_·CH_4_O	*F*(000) = 840
*M**_r_* = 420.08	*D*_x_ = 1.635 Mg m^−^^3^
Monoclinic, *P*2_1_/*n*	Mo *K*α radiation, λ = 0.71073 Å
Hall symbol: -P 2yn	Cell parameters from 2864 reflections
*a* = 11.221 (4) Å	θ = 2.3–24.0°
*b* = 9.642 (3) Å	µ = 2.74 mm^−^^1^
*c* = 15.908 (5) Å	*T* = 298 K
β = 97.537 (5)°	Block, yellow
*V* = 1706.3 (10) Å^3^	0.17 × 0.15 × 0.12 mm
*Z* = 4	

### Data collection

**Table:**

Bruker SMART 1000 CCD diffractometer	3666 independent reflections
Radiation source: fine-focus sealed tube	2345 reflections with *I* > 2σ(*I*)
graphite	*R*_int_ = 0.035
ω scans	θ_max_ = 27.0°, θ_min_ = 2.1°
Absorption correction: multi-scan (*SADABS*; Bruker, 2001)	*h* = −14→14
*T*_min_ = 0.653, *T*_max_ = 0.735	*k* = −12→7
9257 measured reflections	*l* = −20→19

### Refinement

**Table:**

Refinement on *F*^2^	Primary atom site location: structure-invariant direct methods
Least-squares matrix: full	Secondary atom site location: difference Fourier map
*R*[*F*^2^ > 2σ(*F*^2^)] = 0.039	Hydrogen site location: inferred from neighbouring sites
*wR*(*F*^2^) = 0.103	H atoms treated by a mixture of independent and constrained refinement
*S* = 1.02	*w* = 1/[σ^2^(*F*_o_^2^) + (0.0477*P*)^2^ + 0.4564*P*] where *P* = (*F*_o_^2^ + 2*F*_c_^2^)/3
3666 reflections	(Δ/σ)_max_ < 0.001
214 parameters	Δρ_max_ = 0.49 e Å^−^^3^
1 restraint	Δρ_min_ = −0.45 e Å^−^^3^

### Special details

**Table:**

Geometry. All e.s.d.'s (except the e.s.d. in the dihedral angle between two l.s. planes) are estimated using the full covariance matrix. The cell e.s.d.'s are taken into account individually in the estimation of e.s.d.'s in distances, angles and torsion angles; correlations between e.s.d.'s in cell parameters are only used when they are defined by crystal symmetry. An approximate (isotropic) treatment of cell e.s.d.'s is used for estimating e.s.d.'s involving l.s. planes.
Refinement. Refinement of *F*^2^ against ALL reflections. The weighted *R*-factor *wR* and goodness of fit *S* are based on *F*^2^, conventional *R*-factors *R* are based on *F*, with *F* set to zero for negative *F*^2^. The threshold expression of *F*^2^ > σ(*F*^2^) is used only for calculating *R*-factors(gt) *etc*. and is not relevant to the choice of reflections for refinement. *R*-factors based on *F*^2^ are statistically about twice as large as those based on *F*, and *R*- factors based on ALL data will be even larger.

### Fractional atomic coordinates and isotropic or equivalent isotropic displacement parameters (Å^2^)

**Table:**

	*x*	*y*	*z*	*U*_iso_*/*U*_eq_	
Br1	−0.32199 (3)	0.42107 (4)	0.05220 (3)	0.06886 (17)	
Cl1	−0.23673 (8)	0.95108 (9)	−0.05322 (6)	0.0633 (3)	
Cl2	0.49315 (8)	0.21596 (9)	0.18697 (6)	0.0627 (3)	
N1	0.1502 (2)	0.5525 (2)	0.14404 (16)	0.0412 (6)	
N2	0.2713 (2)	0.5514 (3)	0.17335 (16)	0.0421 (6)	
O1	−0.06054 (19)	0.4397 (2)	0.12117 (14)	0.0502 (6)	
H1	0.0113	0.4451	0.1388	0.075*	
O2	0.25707 (19)	0.3476 (2)	0.24068 (15)	0.0588 (6)	
O3	0.1088 (2)	0.2995 (3)	0.36033 (16)	0.0635 (6)	
H3	0.1265	0.3411	0.3188	0.095*	
C1	−0.0179 (2)	0.6676 (3)	0.06979 (17)	0.0372 (7)	
C2	−0.0965 (3)	0.5573 (3)	0.07949 (18)	0.0379 (7)	
C3	−0.2162 (3)	0.5705 (3)	0.04383 (18)	0.0422 (7)	
C4	−0.2581 (3)	0.6892 (3)	0.00215 (18)	0.0467 (8)	
H4	−0.3384	0.6963	−0.0210	0.056*	
C5	−0.1803 (3)	0.7973 (3)	−0.00500 (19)	0.0440 (7)	
C6	−0.0613 (3)	0.7871 (3)	0.02778 (18)	0.0432 (7)	
H6	−0.0094	0.8606	0.0218	0.052*	
C7	0.1096 (3)	0.6595 (3)	0.10313 (18)	0.0416 (7)	
H7	0.1609	0.7323	0.0945	0.050*	
C8	0.3161 (3)	0.4461 (3)	0.22296 (19)	0.0397 (7)	
C9	0.4474 (3)	0.4648 (3)	0.25564 (19)	0.0407 (7)	
C10	0.5333 (3)	0.3652 (3)	0.24473 (18)	0.0428 (7)	
C11	0.6517 (3)	0.3836 (4)	0.2764 (2)	0.0564 (9)	
H11	0.7084	0.3163	0.2683	0.068*	
C12	0.6858 (3)	0.5040 (4)	0.3207 (2)	0.0635 (10)	
H12	0.7661	0.5173	0.3423	0.076*	
C13	0.6033 (3)	0.6032 (4)	0.3332 (2)	0.0596 (10)	
H13	0.6271	0.6831	0.3636	0.072*	
C14	0.4840 (3)	0.5842 (3)	0.3004 (2)	0.0510 (8)	
H14	0.4278	0.6521	0.3085	0.061*	
C15	−0.0087 (4)	0.3311 (6)	0.3718 (4)	0.1076 (17)	
H15A	−0.0086	0.4056	0.4118	0.161*	
H15B	−0.0459	0.2509	0.3928	0.161*	
H15C	−0.0527	0.3587	0.3186	0.161*	
H2	0.313 (3)	0.628 (3)	0.162 (2)	0.080*	

### Atomic displacement parameters (Å^2^)

**Table:**

	*U*^11^	*U*^22^	*U*^33^	*U*^12^	*U*^13^	*U*^23^
Br1	0.0512 (2)	0.0795 (3)	0.0725 (3)	−0.0221 (2)	−0.00436 (17)	0.0152 (2)
Cl1	0.0609 (6)	0.0508 (5)	0.0712 (6)	0.0094 (4)	−0.0180 (4)	0.0155 (4)
Cl2	0.0639 (5)	0.0534 (5)	0.0687 (6)	0.0050 (4)	0.0001 (4)	−0.0110 (5)
N1	0.0336 (13)	0.0412 (14)	0.0463 (14)	0.0017 (11)	−0.0044 (11)	−0.0008 (12)
N2	0.0308 (13)	0.0403 (14)	0.0524 (15)	−0.0008 (11)	−0.0044 (11)	0.0080 (13)
O1	0.0432 (12)	0.0457 (13)	0.0583 (14)	−0.0038 (10)	−0.0059 (11)	0.0127 (11)
O2	0.0429 (12)	0.0537 (14)	0.0781 (17)	−0.0067 (12)	0.0015 (11)	0.0232 (13)
O3	0.0563 (14)	0.0545 (15)	0.0815 (18)	0.0038 (12)	0.0158 (12)	−0.0002 (13)
C1	0.0387 (16)	0.0364 (16)	0.0347 (15)	0.0018 (13)	−0.0019 (13)	−0.0008 (13)
C2	0.0409 (16)	0.0401 (16)	0.0315 (15)	0.0009 (14)	0.0002 (12)	0.0011 (13)
C3	0.0393 (16)	0.0503 (19)	0.0365 (16)	−0.0029 (15)	0.0025 (13)	−0.0018 (15)
C4	0.0357 (16)	0.065 (2)	0.0379 (17)	0.0051 (17)	−0.0002 (13)	0.0001 (16)
C5	0.0471 (17)	0.0391 (17)	0.0434 (18)	0.0091 (15)	−0.0032 (14)	0.0033 (15)
C6	0.0443 (17)	0.0383 (17)	0.0448 (17)	−0.0007 (14)	−0.0024 (14)	0.0006 (15)
C7	0.0361 (16)	0.0420 (17)	0.0438 (18)	−0.0011 (14)	−0.0056 (13)	0.0011 (15)
C8	0.0371 (16)	0.0413 (17)	0.0397 (16)	0.0020 (14)	0.0014 (13)	0.0037 (15)
C9	0.0381 (16)	0.0418 (17)	0.0404 (17)	0.0004 (14)	−0.0020 (13)	0.0077 (14)
C10	0.0435 (17)	0.0456 (17)	0.0376 (17)	0.0022 (15)	−0.0012 (14)	0.0030 (14)
C11	0.0411 (18)	0.064 (2)	0.062 (2)	0.0125 (17)	−0.0019 (16)	0.0058 (19)
C12	0.0423 (19)	0.076 (3)	0.067 (2)	−0.002 (2)	−0.0128 (17)	0.004 (2)
C13	0.055 (2)	0.053 (2)	0.065 (2)	0.0002 (18)	−0.0144 (18)	−0.0064 (18)
C14	0.0508 (19)	0.0451 (19)	0.054 (2)	0.0036 (16)	−0.0054 (16)	0.0038 (16)
C15	0.066 (3)	0.105 (4)	0.155 (5)	0.020 (3)	0.027 (3)	0.001 (4)

### Geometric parameters (Å, °)

**Table:**

Br1—C3	1.883 (3)	C4—H4	0.9300
Cl1—C5	1.749 (3)	C5—C6	1.372 (4)
Cl2—C10	1.735 (3)	C6—H6	0.9300
N1—C7	1.272 (4)	C7—H7	0.9300
N1—N2	1.378 (3)	C8—C9	1.508 (4)
N2—C8	1.343 (4)	C9—C14	1.387 (4)
N2—H2	0.91 (3)	C9—C10	1.388 (4)
O1—C2	1.348 (3)	C10—C11	1.369 (4)
O1—H1	0.8200	C11—C12	1.386 (5)
O2—C8	1.212 (3)	C11—H11	0.9300
O3—C15	1.388 (5)	C12—C13	1.364 (5)
O3—H3	0.8200	C12—H12	0.9300
C1—C6	1.387 (4)	C13—C14	1.383 (5)
C1—C2	1.404 (4)	C13—H13	0.9300
C1—C7	1.461 (4)	C14—H14	0.9300
C2—C3	1.393 (4)	C15—H15A	0.9600
C3—C4	1.374 (4)	C15—H15B	0.9600
C4—C5	1.374 (4)	C15—H15C	0.9600
			
C7—N1—N2	116.8 (2)	O2—C8—N2	123.8 (3)
C8—N2—N1	118.7 (2)	O2—C8—C9	123.5 (3)
C8—N2—H2	125 (2)	N2—C8—C9	112.7 (3)
N1—N2—H2	116 (2)	C14—C9—C10	118.3 (3)
C2—O1—H1	109.5	C14—C9—C8	119.1 (3)
C15—O3—H3	109.5	C10—C9—C8	122.5 (3)
C6—C1—C2	119.7 (3)	C11—C10—C9	121.4 (3)
C6—C1—C7	119.0 (3)	C11—C10—Cl2	118.3 (3)
C2—C1—C7	121.3 (3)	C9—C10—Cl2	120.2 (2)
O1—C2—C3	119.2 (3)	C10—C11—C12	119.0 (3)
O1—C2—C1	122.6 (3)	C10—C11—H11	120.5
C3—C2—C1	118.2 (3)	C12—C11—H11	120.5
C4—C3—C2	121.6 (3)	C13—C12—C11	121.0 (3)
C4—C3—Br1	119.5 (2)	C13—C12—H12	119.5
C2—C3—Br1	118.9 (2)	C11—C12—H12	119.5
C3—C4—C5	119.4 (3)	C12—C13—C14	119.6 (3)
C3—C4—H4	120.3	C12—C13—H13	120.2
C5—C4—H4	120.3	C14—C13—H13	120.2
C6—C5—C4	120.7 (3)	C13—C14—C9	120.7 (3)
C6—C5—Cl1	120.4 (2)	C13—C14—H14	119.6
C4—C5—Cl1	118.8 (2)	C9—C14—H14	119.6
C5—C6—C1	120.4 (3)	O3—C15—H15A	109.5
C5—C6—H6	119.8	O3—C15—H15B	109.5
C1—C6—H6	119.8	H15A—C15—H15B	109.5
N1—C7—C1	119.8 (3)	O3—C15—H15C	109.5
N1—C7—H7	120.1	H15A—C15—H15C	109.5
C1—C7—H7	120.1	H15B—C15—H15C	109.5

### Hydrogen-bond geometry (Å, °)

**Table:**

*D*—H···*A*	*D*—H	H···*A*	*D*···*A*	*D*—H···*A*
O1—H1···N1	0.82	1.86	2.585 (3)	146
O3—H3···O2	0.82	2.04	2.727 (3)	141
N2—H2···O3^i^	0.91 (3)	1.93 (3)	2.830 (4)	176 (4)

Symmetry codes: (i) −*x*+1/2, *y*+1/2, −*z*+1/2.

## References

[bb1] Ali, H. M., Zuraini, K., Wan Jefrey, B. & Ng, S. W. (2007). *Acta Cryst.* E**63**, o1729–o1730.

[bb2] Bruker (2001). *SADABS* Bruker AXS Inc., Madison, Wisconsin, USA.

[bb3] Bruker (2007). *SMART* and *SAINT* Bruker AXS Inc., Madison, Wisconsin, USA.

[bb4] Fun, H.-K., Jebas, S. R., Sujith, K. V., Patil, P. S. & Kalluraya, B. (2008). *Acta Cryst.* E**64**, o1907–o1908. 10.1107/S1600536808028328 PMC2959418 21201117

[bb5] Sheldrick, G. M. (2008). *Acta Cryst.* A**64**, 112–122. 10.1107/S0108767307043930 18156677

[bb6] Zhi, F. & Yang, Y.-L. (2007). *Acta Cryst.* E**63**, o4471.

